# Precise control of miR-125b levels is required to create a regeneration-permissive environment after spinal cord injury: a cross-species comparison between salamander and rat

**DOI:** 10.1242/dmm.014837

**Published:** 2014-04-03

**Authors:** Juan Felipe Diaz Quiroz, Eve Tsai, Matthew Coyle, Tina Sehm, Karen Echeverri

**Affiliations:** 1 University of Minnesota, Department of Genetics, Cell Biology and Development, Stem Cell Institute, 2001 6th St SE, Minneapolis, MN 55455, USA.; 2 Ottawa Hospital Research Institute, Ottowa, ON K1H 8L6, Canada.; 3 University of Erlangen-Nürnberg, Department of Neurosurgery, 91054 Erlangen, Germany.

**Keywords:** Regeneration, Axolotl, Spinal cord injury, microRNAs

## Abstract

Most spinal cord injuries lead to permanent paralysis in mammals. By contrast, the remarkable regenerative abilities of salamanders enable full functional recovery even from complete spinal cord transections. The molecular differences underlying this evolutionary divergence between mammals and amphibians are poorly understood. We focused on upstream regulators of gene expression as primary entry points into this question. We identified a group of microRNAs (miRNAs) that are conserved between the Mexican axolotl salamander (*Ambystoma mexicanum*) and mammals but show marked cross-species differences in regulation patterns following spinal cord injury. We found that precise post-injury levels of one of these miRNAs (miR-125b) is essential for functional recovery, and guides correct regeneration of axons through the lesion site in a process involving the direct downstream target *Sema4D* in axolotls. Translating these results to a mammalian model, we increased miR-125b levels in the rat through mimic treatments following spinal cord transection. These treatments downregulated *Sema4D* and other glial-scar-related genes, and enhanced the animal’s functional recovery. Our study identifies a key regulatory molecule conserved between salamander and mammal, and shows that the expression of *miR-125b* and *Sema4D* must be carefully controlled in the right cells at the correct level to promote regeneration. We also show that these molecular components of the salamander’s regeneration-permissive environment can be experimentally harnessed to improve treatment outcomes for mammalian spinal cord injuries.

## INTRODUCTION

In mammals, axonal regrowth following spinal cord injury is inhibited by the formation of a glial scar consisting primarily of reactive astrocytes and proteoglycans that seem to play important protective roles in stabilizing the delicate central nervous system (CNS) tissue (Sandvig et al., 2004; Silver and Miller, 2004). Injured axons on the rostral or cell-body side of the injury site retract in a process referred to as dieback, and form dystrophic end bulbs that retain the ability to generate new growth cones if given the right environment (Busch et al., 2010). On the caudal side of the injury, the severed axons that are disconnected from their neuronal cell bodies undergo Wallerian degeneration within 24–48 hours, which results in the loss of all motor and sensory function caudal to the injury site (Lubińska, 1982; Kerschensteiner et al., 2005; Wang and Barres, 2012).

Some vertebrates have maintained a remarkable ability to regenerate a fully functional spinal cord after tail amputation or injury (Butler and Ward, 1965; Butler and Ward, 1967; Goss, 1969; Sánchez Alvarado and Tsonis, 2006; Slack et al., 2008; Tanaka and Ferretti, 2009). In the Mexican axolotl salamander, this process involves the formation of terminal vesicle-like structures on both the rostral and caudal ends of the injured neural tube. Cells within ~500 μm of the injury site begin to divide and migrate to replace the missing or damaged portion of the neural tube (Holtzer, 1951; Holtzer, 1952; Holtzer, 1956; Butler and Ward, 1965; Butler and Ward, 1967; Egar and Singer, 1972; Singer et al., 1979; Géraudie et al., 1988). Various genes have been identified that are up- or downregulated in the glial cells at the injured ends of the spinal cord; these cells then proliferate and repair the lesion (Beck et al., 2003; Chernoff et al., 2003; Ferretti et al., 2003; Walder et al., 2003; Chevallier et al., 2004; Schnapp et al., 2005; Monaghan et al., 2007; Moftah et al., 2008; Tanaka and Ferretti, 2009; Dawley et al., 2012; Mchedlishvili et al., 2012; Hui et al., 2013). No evidence of glial-scar formation is detectable in axolotl salamanders, as assessed by pre- and post-injury levels of GFAP, vimentin, chondroitin sulfate proteoglycans (CSPGs) or collagens. Axons regrow across the original lesion site, leading to restored motor and sensory functions caudal to the injury (Clarke et al., 1986; Clarke et al., 1988; Clarke and Ferretti, 1998). It is still unclear how functional recovery occurs in axolotls. It is unclear whether severed axons regrow and reconnect to the same targets, or whether new neurons are born, establishing new neuronal connections.

Many of the glial-scar components that inhibit mammalian axonal regrowth have been identified, and the process of Wallerian degeneration is well understood at the molecular level. However, attempts to promote regeneration after spinal cord injury in mammals are still largely unsuccessful. We have taken a novel approach to understand the molecular cues that regulate gene expression after injury in the regeneration-competent axolotl salamander (*Ambystoma mexicanum*), versus the regeneration-incompetent rat. We focused on the environment of the injury site because the intrinsic differences that occur at the injury site could provide novel insights into how salamanders create a regeneration-permissive environment after injury.

**TRANSLATIONAL IMPACT**

**Clinical issue**

Approximately 265,000 people in the United States currently live with debilitating spinal cord injuries, most of which are the result of car crashes. Current therapies for spinal cord injuries ranging from drugs, electrical stimulations, biomaterial implantations and even stem cell approaches have offered limited success in improving quality of life. To realize the full promise of regenerative strategies in overcoming the natural paucity of axonal regrowth in humans, this field can and must reach beyond mammalian studies by also examining those species, such as the Mexican ‘axolotl’ salamander, that show comprehensive abilities to regenerate complex body structures after injury, a phenomenon that remains poorly understood at the molecular level.

**Results**

In this study, the changes in microRNA (miRNA) levels were examined after spinal cord injury in a regeneration-competent species, the axolotl, versus a regeneration-incompetent species, the rat. A miRNA that is expressed at significantly different levels between the two species, miR-125b, was selected for further *in vivo* analysis. Reduction of miR-125b in the axolotl to levels similar to those in rat was found to inhibit regeneration via upregulation of an ‘axon repulsion’ gene, *Sema4D*. The authors then tested the effect of increasing the levels of miR-125b in rat after spinal cord injury; this was found to result in an overall positive effect on regeneration, including a significant improvement in locomotive ability in some animals.

**Implications and future directions**

The current state of the regeneration and miRNA fields, combined with the models and tools available, offer an excellent opportunity to use comparative studies to determine the molecular and mechanistic basis for the fundamental differences in CNS-healing abilities between non-regenerating mammals and regenerating axolotl. This could provide new insights to advance the development of novel regenerative therapies for major injuries and disabilities in the CNS. This work is one of the few examples of cross-species (axolotl versus rat) comparison after spinal cord injury and has identified key conserved regulatory pathways that react differently in an injury paradigm. The research strategy demonstrated in this work promises a broader impact on regenerative medicine, paving the way for more comparative studies to promote therapeutic regeneration in other organs such as pancreas, heart and liver.

## RESULTS

### microRNA array analysis after spinal cord injury in axolotls and rats

**Fig. 1.**
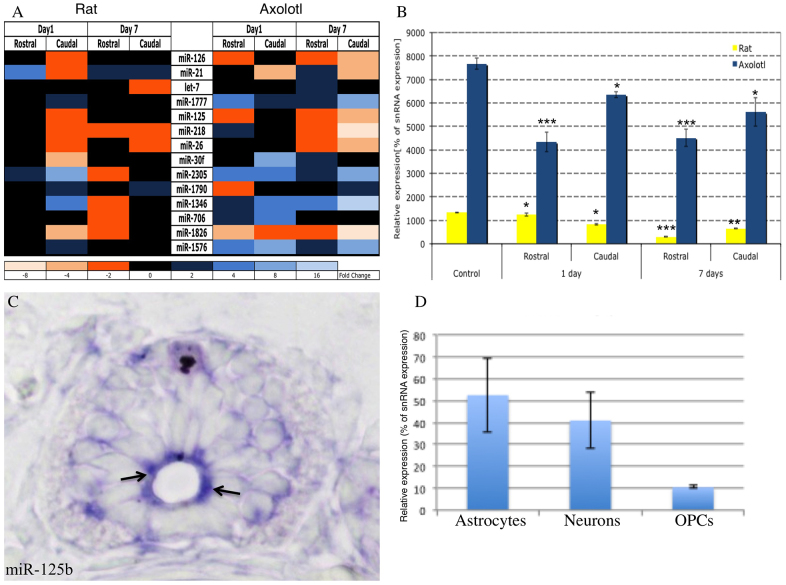
**Conserved miRNAs are differentially expressed in rat versus axolotl after spinal cord injury.** (A) Heat map shows differentially expressed miRNAs at 1 and 7 days after injury, compared with those in uninjured samples. Only miRs with a *P*>0.01 were considered. (B) Quantitative RT-PCR confirms that miR-125b has ~eightfold higher expression in axolotl than in rat in uninjured tissue. It is significantly less abundant in axolotl at 1 day post-injury compared with controls (*P*<0.001), and shows the greatest reduction in levels at 7 days post-injury in rat (*P*<0.001). (C) *In situ* hybridization localizes miR-125b to radial glial cells in the axolotl spinal cord, as indicated by the arrows. (D) Quantitative RT-PCR of miR-125b from individual cultures of primary rat neural cells. OPCs, oligodendrocyte precursor cells. *P<0.05, **P<0.01 and ***P<0.001.

To investigate the molecular basis for the cross-species differences in spinal cord injury repair systems, we focused on microRNAs (miRNAs), which function as high-level pathway regulators and offer convenient entry points for these studies (Hagen and Lai, 2008). Our previous work revealed 93% conservation in miRNAs between axolotl and other vertebrates (Sehm et al., 2009). We initiated the present studies by carrying out a comparative miRNA profiling analysis at different time points following spinal cord transections in axolotl and rat (Fig. 1A). The arrays used covered all known miRNAs from rat, human and mouse at the time of the experiment. Microarray-based miRNA profiles were generated using spinal cord extracts from the rostral and caudal sides of the injury that were collected 1 and 7 days after complete transection of the spinal cord. These time points were chosen so that early changes in miRNA expression could be detected along with those changes that occur later in regeneration when, in axolotl, radial glial cells are dividing and migrating to replace lost tissue and axons are regrowing (Holtzer, 1951; Holtzer, 1956; Butler and Ward, 1965; Egar and Singer, 1972; Singer et al., 1979; Géraudie et al., 1988; Chernoff et al., 2003; Monaghan et al., 2007; Tanaka and Ferretti, 2009), whereas, in rats, a glial scar is taking the place of the lost tissue (Silver and Miller, 2004; Hossain-Ibrahim et al., 2007). We identified miRNAs that exhibited reproducibly significant changes in abundance during the days following the injury, determined whether these changes were specific to only one or both sides of the injury site, and assessed differences within these patterns between the two species (Fig. 1A). The array’s probe collection was designed to cover ~3400 known miRNA sequences from human, mouse, rat and other vertebrate species. Our data identified 27 miRNAs that showed differential regulation at the different time points between the two sides of the injury in rat alone, whereas 36 were significantly differentially changed in axolotl alone. Fourteen miRNAs were identified that showed significant differences after injury in rat versus axolotl (Fig. 1A). This low number of differentially regulated miRNAs suggests that these miRNAs very specifically regulate genes involved in the response to injury in neural tissue. The observed differential regulation of miR-126 suggests that the tissue samples also contained large amounts of vasculature, because expression of this miRNA is well characterized in endothelial cells of zebrafish, mouse and human (Fish et al., 2008; Anand and Cheresh, 2011). When this miRNA is downregulated in zebrafish, it leads to blood-vessel collapse. Because it is downregulated in axolotls after injury, this suggests that quick closure of blood vessels might prevent some inhibitory molecules from arriving at the injury site. This observation could reflect important cross-species differences in angiogenic contributions to the spinal cord repair processes, and it is now undergoing further study.

### Downregulation of miR-125b is necessary for faithful axon regeneration in axolotls

We identified miR-125b as an interesting candidate for further study because it showed marked differences between axolotl and rat, and it has been implicated previously in normal development and in cancer and stem-cell differentiation (Le et al., 2009; Xia et al., 2009; Zhang et al., 2011). Under pre-injury conditions in axolotl, we found that miR-125b expression in the spinal cord was concentrated primarily in radial glial cells (Fig. 1C); the control mismatched probe showed no specific staining (supplementary material Figs S1, 2). Axolotl glial cells have a large nucleus that occupies much of the cytoplasm; therefore, the staining appears at the apical side of the cell adjacent to the central canal. However, the levels in the rat spinal cord were below the detection level of *in situ* hybridization, so qRT-PCR was used to identify the cells that express miR-125b in the adult rat spinal cord. Astrocytes had the highest level of miR-125b expression in rat (Fig. 1C).

In the normal homeostatic condition, miR-125b showed eightfold higher levels in axolotl compared with that in rat (Fig. 1B). By 1 day after spinal cord transection, the rostral side of the injury site showed a rapid ~40% reduction of the initially high miR-125b levels in axolotl, whereas the corresponding levels decreased by less than 1% in rat (Fig. 1B). At the same time point, the caudal side showed a more modest (~20–30%) decrease in miR-125b levels in axolotl, whereas the corresponding levels did not change dramatically in rat. By contrast, the levels of miR-125b in rat displayed marked decreases from day 1 to 7 on both sides of the injury, which coincided with the emergence of a glial scar (Fig. 1B). These data suggest that the earlier and more pronounced decrease in abundance of miR-125b in axolotl compared with rat could be an important and active regulatory process required for regeneration in this species, presumably by triggering the upregulation of one or more gene(s) targeted by miR-125b.

**Fig. 2.**
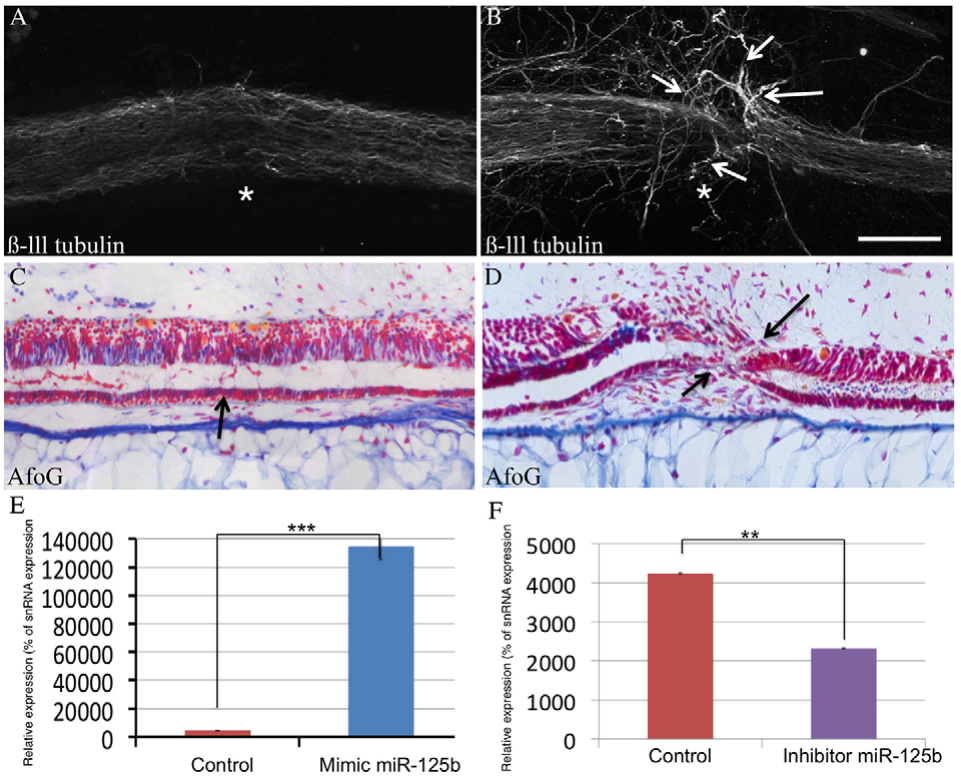
**Increased levels of miR-125b *in vivo* in axolotl after injury leads to defects in regeneration.** (A,B) Whole-mount anti-β-III-tubulin staining in control axolotls (A, *n*=42) versus miR-125b-mimic-injected axolotls (B, *n*=40). Injected axolotls show defects in the regrowth of axons through the lesion site (asterisk); axons appear to sprout in random directions (arrows) but do not grow through the lesion site. (C,D) Longitudinal sections through spinal cord stained with AFOG show a failure of the lumen to reconnect in miR-125b-mimic-treated animals (D, *n*=16) versus controls (C, *n*=20), in which full reconnection of the lumen occurs by 7 days post-injury (arrows). (E,F) Quantitative RT-PCR shows how levels of miR-125b are significantly altered after injection of the mature miR-125b mimic (***P<0.001) (E) or after injecting an inhibitor of miR-125b (**P<0.01) (F). Scale bar: 100 μm.

To test this hypothesis, we studied the effects of modulating miR-125b levels *in vivo* on spinal cord injury repair following complete transection in both axolotl and rat. For the axolotl studies, ablation injuries were performed and the levels of miR-125b were modulated by microinjection of a synthetic inhibitor: an RNA hairpin that binds owing to its complementary sequence to the mature miRNA, sequestering the miRNA and inhibiting the miRNA from functioning. To increase the levels of miR-125b, a commercially available chemically synthesized mature form of the miRNA (mimic) was injected after injury (Diaz Quiroz and Echeverri, 2012). We previously showed that this technique resulted in functionally relevant modulation of miRNA levels during regeneration in axolotls (Sehm et al., 2009). Modulators were microinjected among the glial cells within 500 μm rostral or caudal to the injury because *in situ* hybridization studies showed that these were the main cells expressing miR-125b (Fig. 1C). The optical transparency of the axolotl allows us to clearly identify where the lumen of the spinal cord is and to therefore specifically inject into the central canal or into groups of glial cells. Electroporation was used to aid uptake of the mimics or inhibitors (Diaz and Echeverri, 2012). The efficiency of modulation of the levels of miR-125b was quantified by qRT-PCR, and was effective for both mimic and inhibitor (Fig. 2E,F). The mimic induced increased levels of miR-125b, resulted in aberrant sprouting of the axons on both the rostral and caudal sides, and reduced the growth of axons through the lesion site by 7 days post-injury (Fig. 2B), at which time the control animals showed full regeneration (Fig. 2A). Histological examination of the underlying neural tube revealed that the reconnection process remained incomplete despite clear regrowth from both sides, and resulted in a failure to restore an open lumen, compared with control animals, which showed full reconnection (Fig. 2C,D). The aberrant axon sprouting was observed at exactly the point where the rostral and caudal sides of the neural tube failed to reconnect, consistent with the possibility that either the re-growing axons use the neural tube as a directional guide or that reconnection of the tube might require correctly oriented axons.

**Fig. 3.**
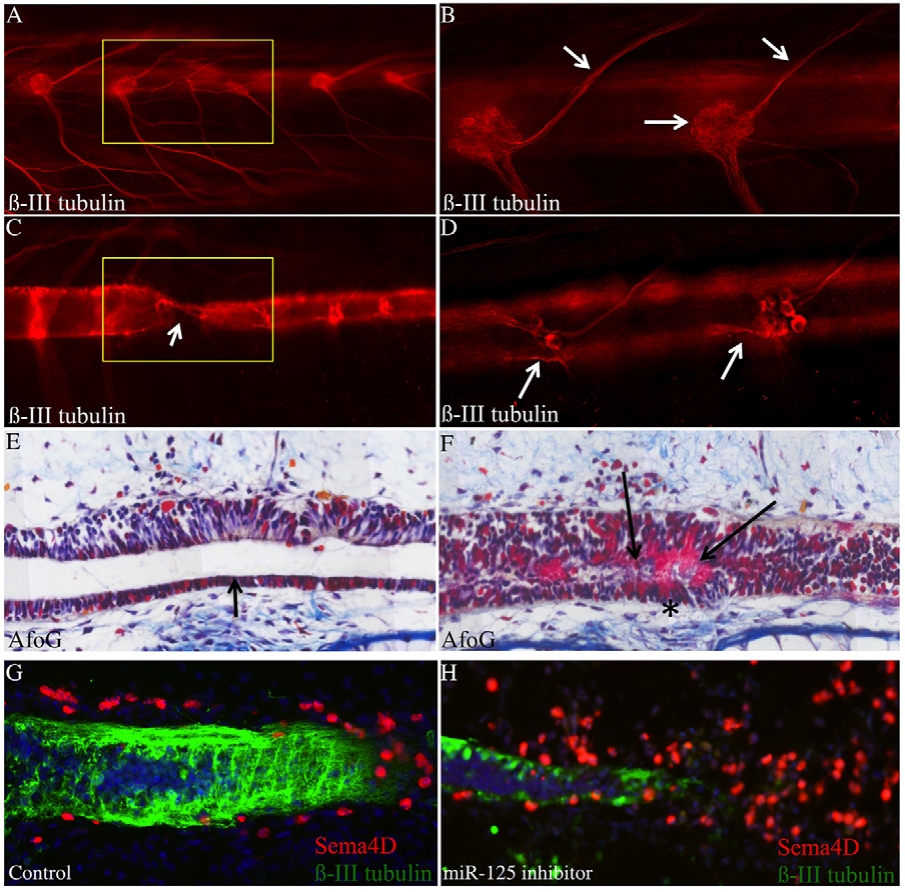
**Inhibition of miR-125b in axolotl after injury causes defects in regeneration.** (A–D) Whole-mount anti-β-III-tubulin staining of control (A,B) (*n*=48) and miR-125b-inhibitor-treated (C,D) (*n*=51) axolotls. Upon inhibitor treatment, axons fail to cross the injury site (yellow box and arrow in C), and degeneration of dorsal-root ganglion axons is observed on the caudal side of the injury (arrows D), whereas this is never observed in controls (A,B). Yellow box marks the injury site. Arrows in B indicate normal dorsal-root ganglion cell bodies and axons. (E,F) AFOG histological staining of sections. In control-injected animals, rostral and caudal sides of the neural tube are reconnected (arrow, E) (*n*=10). In miR-125b-inhibitor-injected animals, fibrin deposition, an indicator of scar tissue, is observed in the neural tube (arrows and asterisk in F) (*n*=15). (G,H) *Sema4D*, a predicted target gene of miR-125b, is normally found in cells on the outside of the neural tube during axonal regeneration (G) (*n*=12); by contrast, in animals treated with miR-125b inhibitor, Sema4D protein is found in many more cells throughout the injury site (H) (*n*=15). Green: anti-β-III-tubulin; red: anti-Sema4D; blue: DAPI.

Microinjection of a miR-125b inhibitor in axolotl to reduce miR-125b to levels similar to those observed in rats resulted in strong inhibition of axonal regeneration (Fig. 3A,C) and a degeneration of axons caudal to the injury site (Fig. 3B,D). Regeneration of the neural tube was complete in this case, including full reconnection and restoration of the open lumen, although with a notable deposition of fibrin identified by staining with acid fuchsin orange G (AFOG) (Fig. 3E,F). This result is reminiscent of the glial scar observed in non-regenerative organisms. These data indicate that precise control of miR-125b expression is required for correct regeneration of the axolotl spinal cord following complete-transection injuries. The phenotypic observations from the inhibition of miR-125b and from increasing the levels of mature miR-125b after spinal cord injury suggest that a precise regulation of that miRNA is necessary to achieve functional regeneration in axolotl.

### *Sema4D* is a conserved target of miR-125b in axolotl and rat

We used bioinformatics analyses to identify downstream protein-coding transcripts regulated by miR-125b. These studies identified the well-known class of Semaphorin genes, in particular *Sema4D*, as a predicted target of this miRNA. *Sema4D* has been described previously as a transmembrane axonal repulsion cue whose expression is upregulated in mice at the site of injury following spinal cord injury (Moreau-Fauvarque et al., 2003). In uninjured axolotl, Sema4D protein was not detectable by immunofluorescence in glial cells, whereas it was observed in rat GFAP+ astrocytes (supplementary material Fig. S1A,B). To test whether this pathway is conserved in axolotl, we cloned the axolotl homolog of *Sema4D* and found that it contained an 8-mer seed sequence for miR-125b in its 3′ UTR (supplementary material Fig. S1B). We confirmed that the 3′ UTR of axolotl and rat *Sema4D* could confer downregulation of a reporter luciferase gene expressed in cells in culture (supplementary material Fig. S1D).

*Sema4D* expression in cells on the outside of the neural tube was transiently upregulated in axolotl at 3 days post-injury. This suggests a possible role in guidance of the re-growing axons through the lesion site (Fig. 3G). This role for *Sema4D* in axolotl, and its regulatory link to miR-125b, were both corroborated by results showing that inhibition of miR-125b causes upregulation of *Sema4D* in cells at the injury site (Fig. 3H; supplementary material Fig. S3A), whereas decreased levels of *Sema4D* were observed in mimic-treated animals (supplementary material Fig. S3B).

On the basis of these results, we hypothesize that the requirement for precisely controlled levels of miR-125b early during axolotl spinal cord regeneration underlies an equally precise and rapid increase in expression of *Sema4D* that is essential for faithful and functional regeneration. In support of this model, overexpression of either axolotl or human *Sema4D* in cells at the injury site was sufficient to inhibit axonal regeneration in axolotl, and generates a similar phenotype as that resulting from treatment with the miR-125b inhibitor (supplementary material Fig. S3A).

We investigated how these results might translate to the mammalian model by modulating the levels of miR-125b *in vitro* in rat primary neural cells. Initial cell-culture experiments showed that astrocytes had the highest levels of both miR-125b and *Sema4D* (Fig. 1D; supplementary material Fig. S1B). *In vitro* assays confirmed that a reduction in miR-125b levels in astrocytes leads to an increase in *Sema4D* levels (supplementary material Fig. S4B). We then examined the effects of decreased levels of *Sema4D* in astrocytes on the neuronal response to scratch-induced injury. When neurons were grown on astrocytes under normal *in vitro* conditions, the axons retracted and did not grow into the injury site (supplementary material Fig. S5A,C). Depletion of Sema4D protein in astrocytes by RNAi created a more permissive environment, and the injured neurons projected axons into the scratched area (supplementary material Fig. S5B,D). Using this cell-culture injury model, we observed that *in vitro* inhibition of miR-125b in astrocytes led to increased levels of Sema4D (supplementary material Fig. S4A,B). These results confirm that this regulatory pathway is conserved in axolotl and rat.

### Increased levels of miR-125b promote a more regeneration-permissive environment in rat

**Fig. 4.**
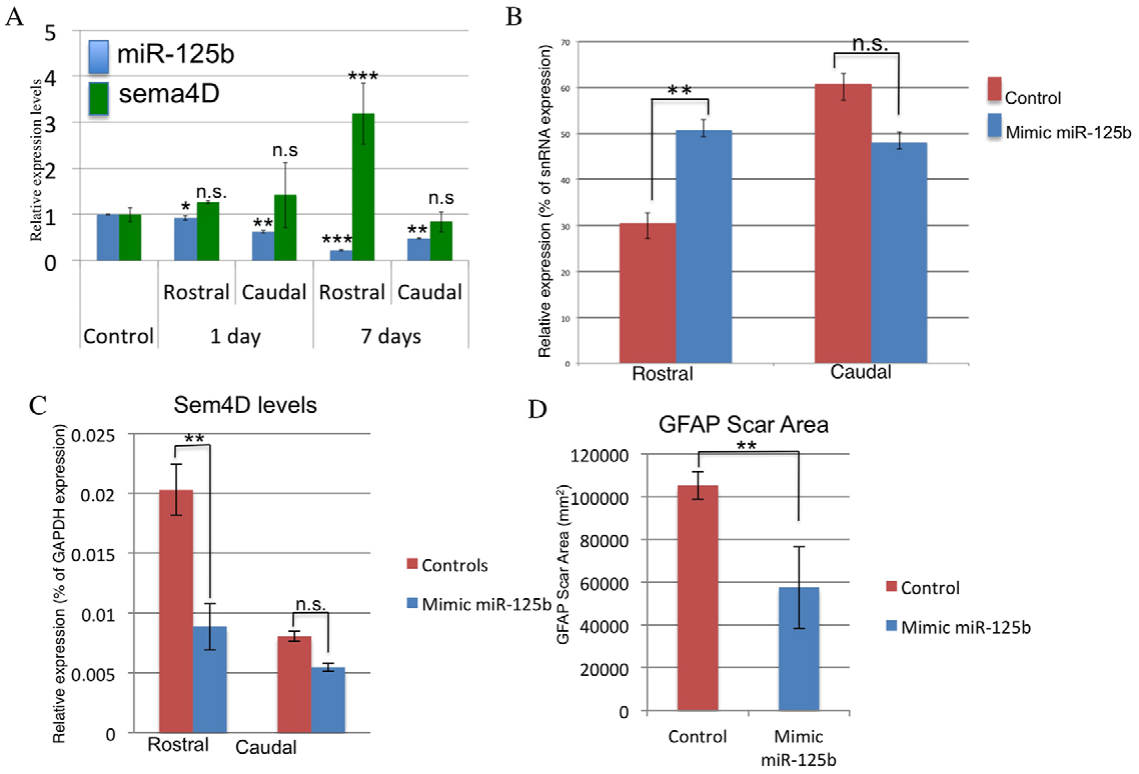
**Mimic treatments to increase miR-125b levels *in vivo* following spinal cord injury in rat lead to decreased levels of the target gene *Sema4D*.** (A) Untreated animals show a decrease in abundance of miR-125b (blue bars) *in vivo* by 7 days post-injury, whereas endogenous levels of Sema4D (green bars) are increased by 7 days post-injection. (B,C) Local post-injury treatment with a synthetic mimic of miR-125b leads to a significant increase in miR-125b on the rostral side of the injury (B), whereas reduced levels of *Sema4D* are observed (C). (D) Post-injury modulation of miR-125b causes a significant decrease in the size of the GFAP-positive glial scar tissue. *P<0.05, **P<0.01 and ***P<0.001.

We tested the effect of modulating the levels of miR-125b *in vivo* in rat following spinal cord injury. After spinal cord injury in rat, miR-125b levels were significantly decreased by 7 days post-injury and *Sema4D* levels increased (Fig. 4A). A synthetic mimic of the mature form of rat miR-125b, which is identical to human, mouse and axolotl miR-125b, was mixed into pluronic gel, an inert biodegradable gel that facilitates the localized delivery of siRNAs *in vivo* to the spinal cord (Cronin et al., 2006). The miR-125b in pluronic gel was injected into the lesion site immediately after injury. At 7 days post-injury, tissue samples showed a significant increase in miR-125b levels on the rostral site of the injury compared with that of control (Fig. 4B), and had decreased levels of *Sema4D* (Fig. 4C). These results support the proposed miR-125b–*Sema4D* regulatory pathway.

**Fig. 5.**
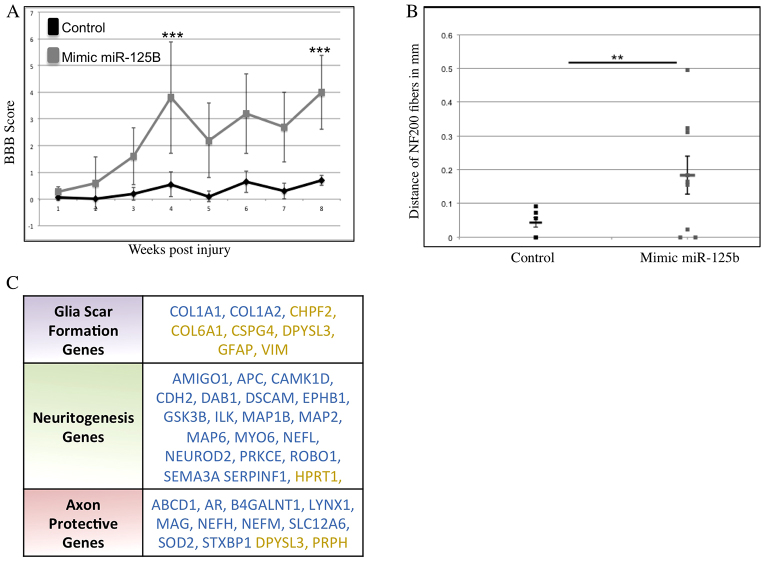
**Mimic treatment to increase miR-125b levels *in vivo* in rat following complete spinal cord transection improves functional recovery.** (A) Results from open-field locomotion experiments (cohort size of *n*=13) revealed significant improvements in BBB scores, ranging up to 8, in animals treated with the miR-125b mimic (***P<0.001). (B) Axons grew further into the scar tissue area in miR-125b-mimic-treated animals compared with controls (**P<0.01). (C) Array analysis of miR-125b-mimic-treated animals showed that glial-scar genes, neurogenesis genes and axon-protective genes displayed the most notable differential regulation after treatment. Blue text indicates genes that were upregulated in miR-125b-mimic-treated rats; yellow text indicates genes that were downregulated in miR-125b-mimic-treated rats.

Animals were assessed weekly for detectable changes in locomotive abilities using the Basso, Beattie and Bresnahan (BBB) scoring method (Koopmans et al., 2005; Martinez et al., 2009). These tests demonstrated significant improvements in the miR-125b-treated animals compared with controls. This included BBB scores ranging from 4–5 for the majority of miR-125b-mimic-treated animals. Some animals achieved scores of 6–8, accounting for the sizeable standard deviation from the mean at 4 weeks post-treatment, which represents remarkable recoveries from complete spinal cord transection (Fig. 5A). Effects on bladder control were also assessed; however, no significant differences were observed. At 8 weeks, the animals were euthanized and effects on glial-scar formation and axonal outgrowth were examined. Mimic-treated animals with BBB scores of 4–8 showed clear decreases in the levels of *GFAP* expression, and overall decreases in the size of the GFAP+scar tissue (Fig. 4D; supplementary material Fig. S6). In the miR-125b-treated animals, the number and length of axons projecting into the scar tissue increased (Fig. 5B). However, *in vivo* antero- and retrograde tracing of axons in control- and mimic-treated animals showed no clear evidence of regeneration of long-tract axons in miR-125b-mimic-treated animals. This suggests that the axons in the injury site observed by histological staining are possibly regrowth of local interneurons.

**Fig. 6.**
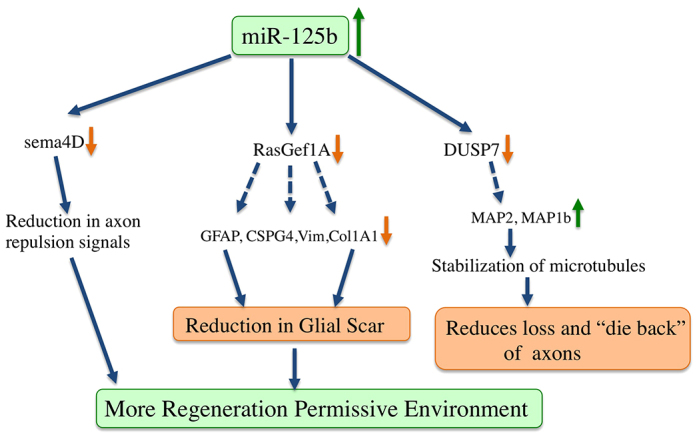
**Model of how miR-125b promotes a regeneration-permissive environment in rat.** Array analysis identified differential regulation of multiple targets of miR-125b in the mimic-treated rat samples. The phenotype observed is due to modulation of several pathways, possibly due to reducing production of several glial-scar genes, creating a less hostile tissue for axons to regrow on. Significant regulation of genes involved in stabilizing microtubules was also observed, suggesting that more axons might be preserved at the injury site in miR-125b-treated animals.

Because miRNAs can target hundreds of genes, it is unlikely that the phenotypic effects resulting from the mimics are due to modulation of *Sema4D* alone. Therefore, microarrays were performed using RNA samples harvested at 1 week post-treatment of control- and miR-125b-mimic-treated animals. The arrays revealed ~1500 genes that were differentially regulated in the mimic-treated animals; 53 of these were predicted to have seed sequences for miR-125b in the 3′ UTR, and 23 of the 53 were significantly decreased on the rostral side of the injury (supplementary material Table S1). Genes involved in glial-scar formation were affected, as observed in the significantly diminished levels of *GFAP*, *CSPG4* and *Col6A1* (Fig. 5C) in mimic-treated versus control samples. The arrays identified significantly higher levels of genes involved in microtubule stabilization, neurite outgrowth and axon guidance in mimic-treated rats (Fig. 5C). These data suggest that the mimic treatment improves the preservation of severed axons on the rostral side of the injury and promotes an environment that is more favorable to post-injury neurite outgrowth by inhibiting the expression of some glial-scar proteins (Fig. 5C; Fig. 6).

## DISCUSSION

This study shows that regulators of gene expression are conserved among vertebrates, but can exhibit very different responses to injury. miR-125b is expressed in similar cell types in axolotl and rat, but the expression levels are very different in uninjured and injured tissues. It is unknown what regulates the miR response after injury, and regulation might occur at many different levels because the response of cells to injury is extremely complex.

**Fig. 7.**
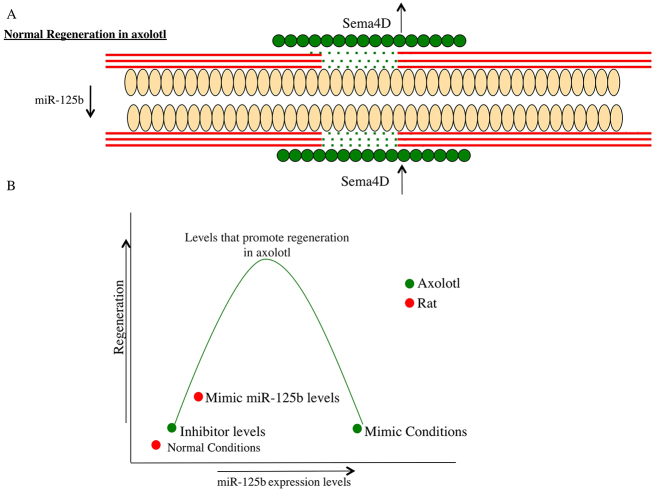
**Model of miR-125b and *Sema4D* regulation of axonal regeneration in axolotl after injury.** (A) Schematic diagram of the axolotl spinal cord undergoing regeneration. Peach ovals represent the radial glial cell, red lines are the axons and the green circles represent cells that express Sema4D protein after injury. miR-125b levels are reduced after injury, whereas *Sema4D* is more abundant in a discrete set of cells (green circles) and functions to repel axons (red lines) and thereby guide them to grow through the lesion site (green dots) along the ependymal tube formed by radial glial cells (peach ovals). (B) The *in vivo* axolotl experiments show that, if miR-125b levels are too high or too low, it leads to defective axonal regeneration. This suggests that a balance of miRNA and target gene expression is necessary for perfect regeneration.

We used established methods for modulating gene expression levels *in vivo* in axolotl, and showed that inhibition of miR-125b leads to defective axonal regeneration due to upregulation of the axon-repulsion gene *Sema4D*. Maintaining high levels of miR-125b after injury also leads to defective axonal regeneration because the axons sprout randomly around the injury site and are not directed through the lesion site. Our data show that miR-125b and its target *Sema4D* need to be regulated in the right cells at the right time to guide axons through the lesion site (Fig. 7A,B). This suggests that miR-125b plays a role in fine-tuning gene expression during regeneration (Fig. 7B). We do not rule out that miR-125b could target other genes that might be involved in the glial-scar response. The inhibitor-treated cells did show an increase in fibrin deposition, which is indicative of scarring. In previous studies we also used transcriptional profiling to identify miRNAs involved in the early stages of tail regeneration; interestingly, in that study, increased levels of miR-125b were seen at 3 days post-amputation (Sehm et al., 2009). This could suggest that miR-125b plays a different role depending on the regeneration paradigm and will be interesting to determine in the future.

We then examined the role of miR-125b in rat. The initial comparative array analysis showed that miR-125b is expressed at ~eightfold lower levels in rat than in axolotl. After injury, only a slight decrease in miR-125b was initially observed, but its levels were significantly reduced at 7 days post-injury, when the predicted target gene, *Sema4D* levels are increased. *In vitro* analysis in primary astrocytes showed that inhibition of miR-125b leads to increased levels of the Sema4D protein, and reduction of Sema4D by RNAi creates a more permissive environment for neural outgrowth after scratch-induced injury (supplementary material Fig. S5). These data gave us a rationale to modulate miR-125b levels *in vivo* in rat after spinal cord injury. This was achieved by mixing the chemically synthesized mature form of the miRNA into a biodegradable gel and directly injecting it into the injury site. Previous work showed that cells internalize the gel via endocytosis, and thereby take up the gel contents. This strategy worked for performing RNAi *in vivo* (Cronin et al., 2006; Cronin et al., 2008) and for delivery of the miRNA in the current study. The qRT-PCR analysis showed that the method was successful for delivering the mimic, resulting in a decrease in the predicted target gene on the rostral side of the injury, but no effect was observed on the caudal side of the injury. This might be because the gel was not taken up by those cells, or because it was displaced from that area, or because, by the time of analysis, those cells might have died. Despite this, we observed a clear improvement in the locomotive abilities of the mimic-treated animals compared with that in control-treated animals after complete transection. Histological analysis showed a decrease in *GFAP* expression in the scar area and an increase in axons growing into the scar tissue, which suggests a more regeneration-permissive environment. Although anterograde and retrograde tracing of axons was attempted, the results were inconclusive owing to the high background in the samples in the scar-tissue area. This leaves an open question as to whether the improved locomotive ability is due to regeneration of long-tract cortical axons, prevention of dieback of the severed axons, or to a more local regeneration of inter-neurons. Behavioral analyses conducted in *Sema4D*-knockout mice showed increased motor activity when subjected to the open-field locomotive test compared with that of control mice, suggesting that a lack of *Sema4D* might promote more neurogenesis (Yukawa et al., 2009). This observation in the knockout mice corresponds with the locomotive recovery observed in the miR-125b-treated rats.

The array analysis of the animals shows that a single dose of miR-125b targets multiple pathways that improve functional recovery after complete transection of the spinal cord. In the future it will be interesting to follow up on the role of several of these target genes; for example, *SLC16A6*, which encodes a monocarboxylate transporter, is a predicted target of miR-125b and its expression levels were significantly decreased in miR-125b-mimic-treated rats after injury. Previous work in *Xenopus* limb and tail regeneration has linked other monocarboxylate transporters, such as SLC5A8, to regeneration-induced epigenetic modifications. It will be interesting to determine whether modification of these transporters can play a similar role in spinal cord injury repair in mammals (Tseng et al., 2010; Tseng and Levin, 2012). This result opens many new avenues for locally targeted treatment of spinal cord injury using a method that does not cross the blood-brain boundary. In the future, it will be essential to test whether multiple treatments with miRNA alone increase the functional recovery, or whether combinations of modulators of miRNAs are more effective.

Our data define the first miRNA-regulated pathway involved in axolotl spinal cord injury repair, and elucidate key functional components of this pathway. This comparative study offers the first substantial translation of new molecular insights aimed at defining a new biological understanding of major mammalian pathways and new avenues for the development of innovative treatments for human spinal cord injuries.

## MATERIALS AND METHODS

### Axolotl surgery

Adult axolotls were used for these experiments. Animals were anesthetized using 0.1% benzocaine (Sigma). An incision was made in the skin above the position of the hind limb, a portion of the skin and muscle was removed, and then a laminectomy was performed. A 2-mm portion of the exposed spinal cord was removed. The skin was replaced over the wounded site and the animals were placed back into water. In control animals, only a laminectomy was performed. A similar procedure was performed at 1 and 7 days post-injury, and the tissue of the injury site plus 1-mm of tissue rostral and caudal to the injury site was collected and placed immediately into liquid nitrogen.

### Comparative miRNA array analysis

Total RNA was extracted from the tissue samples using the Trizol protocol (Invitrogen). Triplicates of each time point were carried out. For rat and axolotl, pooled tissue from ten adult animals was used for each sample time point. An miRNA array containing 3361 probes of miRNAs expressed in vertebrates (Vertebrata miRNA Array, MRA-1033C, LC Sciences, USA) was performed. The results were measured as average signals, which were filtered using an average signal of 500 as a baseline. Then, the filtered data for each miRNA were expressed for each condition (rostral or caudal, 1 or 7 days post-injury) as a fold-change (FC) on a log_2_ scale as follows:

$$\text{FC}=\log_{2}(\frac{\text{Av.Exp.Cond.}}{\text{Av.Control}}),$$

where Av.Exp.Cond. = average signal of the experimental condition, and Av.Control = average signal of the control condition.

Data were processed by first subtracting the background using regression-based mapping, and then normalizing the signals using a locally-weighted scatter plot smooth (LOWESS) filter. For a two-color experiment, the ratio of the two sets of detected signals (log_2_ transformed, balanced) and *P*-values of the *t*-test were calculated. Differentially detected signal sets with *P*-values below 0.01 were considered as statistically significant. To facilitate the analysis of the array data, identical miRNAs of different species (e.g. bta-miR-126 and hsa-miR-126) were grouped together with miRNAs with the same precursor or closely related mature sequences (e.g. mdo-miR-26, hsa-miR-26a), as long as the seed sequences were still conserved. Then, for each system (rat and axolotl), the numbers of miRNAs that displayed changes in expression in response to injury were quantified, and miRNAs that change expression in both systems were identified. For miRNAs that were differentially expressed in both systems (14 in total), the predicted targets were determined using open-source bioinformatics software (TargetScan, PicTar and microRNA.org).

### *In vivo* modulation of miR-125b and *Sema4D* levels in axolotls

All the animals used in the experiments were naturally bred and grown in the axolotl facility of the Max Plank Institute for Cell Biology and Genetics (Dresden, Germany) and the University of Minnesota. At day 0, axolotls with lengths of 3–5 cm were anesthetized by submersion in 0.01% benzocaine (Sigma). Animals received injections into the spinal cord with 10 μM miR-125b mimic or 20 μM miR-125b inhibitor (Dharmacon, Thermo Scientific) suspended in buffer [phosphate-buffered saline (PBS) + 0.1 mg/ml Fast Green] using a pressurized microinjector (WPI) and a Leica dissecting microscope. To facilitate the uptake of mimic or inhibitor, the injection site was electroporated (5 square pulses, 50 ms, 50 V) twice. Animals that were injected with 10 μM control mimic (Dharmacon, Thermo Scientific) plus Fast Green were used as controls. At 30 minutes to 1 hour after the injections, the skin and muscles above the spinal cord (including 7–10 muscle bundles caudal to the hind limbs) were removed under the dissection microscope. An ablation injury was performed by removing a portion of the spinal cord (approximately 300 μm) as described previously (Diaz and Echeverri, 2012). After the injury was performed, the animals were put back in water. At 2 and 4 days after the injury, the animals were re-injected with miRNA mimic or inhibitor into the spinal cord at a distance of 5 muscle bundles rostral and caudal from the injury site. A similar procedure was carried out to overexpress the full-length axolotl, rat or human Sema4D at the injury site in axolotl after injury. The ubiquitous cytomegalovirus (CMV) or CAGGS promoter was used to drive expression of the full-length *Sema4D* gene.

### Whole-mount staining

At 7 days after injury, tissue samples were collected by amputating the tail of anesthetized axolotls at the level of the hind limb. The tip and fins of the tail were removed, and the tissue samples were fixed in freshly made 4% paraformaldehyde (PFA) (Sigma) in PBS for 1 hour. To improve the antibody penetration of the tissue, samples were washed three times with 0.1% Tween-20 (Sigma) in PBS (PBST) for 5 minutes, digested with 20 μg/ml proteinase K in PBS for 30 minutes, and post-fixed in 4% PFA for 10 minutes. The samples were washed with PBST three times for 5 minutes, and then washed three times with 0.2% Triton X-100 (Sigma) in PBS (PBSTX) for 10 minutes. To prevent non-specific binding of the antibodies, the samples were blocked in 10% goat serum (Sigma) in PBSTX for 1 hour at room temperature. Mouse anti-β-III-tubulin primary antibody (Sigma) was diluted 1:1000 in blocking buffer, and samples were incubated in the antibody solution overnight at 4°C. The next day, unbound antibody was eliminated by washing the tissue samples four times in PBST for 30 minutes. The secondary antibody (goat anti-mouse Alexa Fluor 568, Invitrogen) and DAPI were both diluted to a final concentration of 1:200 in blocking buffer, and the samples were incubated in the secondary antibody for 1 hour at room temperature. To eliminate the unbound antibody, samples were washed four times in PBST for 30 minutes. Prior to imaging, samples were cleared with 1:2 benzyl alcohol:benzyl benzoate (BABB) (Sigma). Tissue samples were dehydrated by incubating in 50:50 PBS:methanol for 10 minutes and 100% methanol twice for 10 minutes. The samples were then incubated in BABB for 10 minutes, and mounted onto a coverslip using BABB as the mounting medium. Images were captured using an inverted Zeiss Apotome Microscope (Carl Zeiss) at 10× magnification. All incubations and washes were carried out at room temperature unless indicated otherwise.

### AFOG staining

At 7 days after injury, tissue samples were collected as described and fixed in freshly made 4% PFA with 0.01% glutaraldehyde (Sigma) in PBS for 3 hours. After fixation, samples were washed three times in PBS for 5 minutes and in PBST three times for 5 minutes. Then, tissue samples were incubated for 10 minutes in 50:50 PBS:30% sucrose in PBS, and overnight in 30% sucrose in PBS, before embedding the samples in Tissue Tek (Sakura). Frozen tissue samples were cut into 20-μm-thick longitudinal sections. Tissue sections were post-fixed in Bouin’s solution (Sigma) overnight and washed with running double-distilled H_2_O (ddH_2_O) for 30 minutes. Then, the sections were stained by successive 5-minute incubations in 1% phosphomolybdic acid (Sigma), AFOG solution [0.5% aniline blue (Waldeck-Chroma), 1% orange G (Fluka), 1.5% acid fuchsin (Sigma)], and 0.5% acetic acid. The slides were washed with running ddH_2_O for 5 minutes in between the incubations with stain solution. After the staining step, the slides were dehydrated by successive incubations in 96% ethanol for 2 minutes, 100% ethanol for 2 minutes and xylene for 5 minutes, before mounting with Cytoseal 60 (Thermo Scientific).

Images were captured using a Zeiss Apotome Microscope (Carl Zeiss) at 20× magnification.

### Semaphorin 4D cloning

To clone the Semaphorin 4D (*Sema4D*) gene of axolotl, primers were designed to target conserved regions in the sequences coding the Sema domain in different species. The final sequence of the primers was established using expressed sequence tags (ESTs) of the *Sema4D* of axolotl as follows: S4D_472_fwd 5′-TTCCACTCAGACATCAGCTTCC-3′; S4D_1107_rev 5′-CAAAAGTTGAATATACCCAAAGGG-3′.

RT-PCR (One-Step RT-PCR kit, Qiagen) was performed using total RNA extracted from spinal cords of axolotls injected with 20 μM miR-125b inhibitor. The PCR product was cloned into a p-Gem-T-Easy vector for sequencing (pS4D). To clone the 5′ and 3′ ends of *Sema4D*, new primers were designed using the sequence obtained with the previous primers as follows: S4DRACE_510_fwd 5′-CTGAGACAGAATGCGGGAATGGCAATAT-3′; S4DRACE_91_rev 5′-GGTTAGCGGTACTGTCGGGGAAGCTGAT-3′.

Rapid amplification of cDNA ends (RACE)-PCR (Invitrogen) was performed using 5′ and 3′ RACE-cDNA synthesized from the total RNA that was extracted previously.

### Rat surgeries

All animal procedures were approved by the animal care committee of the Ottawa Hospital Research Institute (OHRI) in accordance with regulations established by the Canadian Council on Animal Care (CCAC). Female Sprague-Dawley rats weighing 250–300 g (Charles River, St Constant, Quebec) were used for this study. The rats were maintained under anesthesia with 2–5% Isoflurane, and preoperatively received 3 ml of subcutaneous normal saline. Sterile ointment was applied to the eyes. The rats were then shaved and the skin was prepared with povidone iodine (Betadine). With the assistance of a surgical microscope (Zeiss), a midline incision was made in the skin, and the paraspinal muscles were dissected from the T7–T9 spinous process and lamina. The spinal cord was exposed and a T7-T9 laminectomy was performed using 6-mm serrated malleus nippers (Storz). For those animals receiving transections, microscissors were used to completely transect the spinal cord at the T8 level, and subsequent bleeding was controlled with Surgifoam (Ethicon). To ensure complete transection, a glass hook was passed around the ventral aspect of the cord into the transection site to ensure that all tissue connections were ablated. We also carefully inspected the transection site to ensure there was no tissue that was spared from transection. For the miRNA analysis, animals received either a sham operation of laminectomy only (control), or a laminectomy with a complete transection. For each group, animals underwent survival of 1 day (*n*=10/group) or 7 days (*n*=10/group). To assess whether miRNA supplementation affected spinal cord repair, another set of animals underwent spinal cord transection as described above, and underwent treatment with 100 μl pluronic gel alone (*n*=14) or pluronic gel with 10 μM miR-125b (*n*=18) applied into the transection site. Gel was allowed to set for 1 minute, and then a section of Duraform (DePuy) was placed over the rostral and caudal stumps, covering the transection site and implanted gel. The surgical site was then closed in layers using 3-0 vicryl sutures (Johnson & Johnson, Peterborough, Ontario, Canada) to approximate the muscles, and 9-mm Michel suture clips (Fine Science Tools) to approximate the skin. Rats were revived from anesthesia with O_2_, given an immediate post-operative injection of buprenorphine (Temgesic) at a dose of 0.05 mg/kg body weight subcutaneously, and were monitored closely while under a heat lamp. Rats underwent bladder expression three times a day. Urinary tract infections were treated with a course of 100 mg/kg body weight ampicillin, which was given twice daily for a period of 5 days.

### Pluronic gel

A 15% (w/v) solution of pluronic gel was made by heating sterile DNase/RNase-free PBS to 90°C and slowly mixing in the pluronic (Sigma) powder. Once all powder was dissolved, the magnetic stirring plate and gel were placed at 4°C until implantation. Immediately prior to surgery, miR-125b mimic (Qiagen) was mixed with the 15% pluronic gel at 4°C to obtain a final concentration of 10 μM.

### Behavioral analysis

Both groups that received pluronic gel and pluronic gel with miR-125b mimic underwent behavioral assessments once a week for 8 weeks. Functional assessment was performed with the Basso, Beattie and Bresnahan (BBB) scoring system (Basso et al., 1996). All animals were videotaped while ambulating for 4 minutes.

### Tissue preparation

Animals whose tissue was to be used for miRNA analysis were anesthetized deeply with Isoflurane and euthanized with a 500-ml intracardiac perfusion of 10 mM ice-cold PBS. Spinal cords were exposed and both rostral and caudal stumps (2 cm in length) were dissected. Tissue segments were flash-frozen in liquid nitrogen for ~5 minutes and stored at −80°C for later miRNA analysis. For the long-term regeneration experiments, animals were euthanized as described above, and tissue was perfused with freshly made ice-cold 4% paraformaldehyde (Sigma). CNS tissue was removed and post-fixed overnight in 4% PFA. Tissue was then transferred to a 20% sucrose solution in PBS for storage until used for sectioning. Tissue segments were embedded in OCT (Tissue-Tek) and frozen. Coronal sections of the entire brain were cut into 40-μm sections using a cryostat. Serial 20-μm parasagittal sections of the spinal cord transection site were obtained and mounted on Superfrost-Plus slides (Fisher Scientific, Markham, ONT, Canada). Sections for immunohistochemistry were kept frozen at −20°C and processed for immunohistochemistry shortly following sectioning.

### Immunohistochemistry

Standard immunofluorescence techniques were used to assess the transection site. The following primary antibodies were used: mouse anti-neurofilament 200 (NF200, 1:500, Sigma) and rabbit anti-glial fibrillary acidic protein (GFAP, 1:200, Millipore). All sections were washed three times with PBS for 10 minutes, and blocked/permeabilized with 10% normal goat serum (NGS) in 0.3% Triton X-100 in PBS for 45 minutes. Sections were washed an additional three times with PBS and incubated overnight at 4°C with primary antibodies diluted in 10% NGS PBS. Then, sections were washed three times and incubated at room temperature in PBS containing Alexa Fluor 594 goat anti-mouse for NF200 and Alexa Fluor 488 goat anti-rabbit for GFAP. Sections were washed a final three times and coverslips were mounted with mounting media (Vector Laboratories, Burlingame, CA).

### Image analysis

Confocal images were obtained using a Nikon Ti Eclipse C1 system. Briefly, sections containing the central canal (CC) were chosen for image analysis; 20-μm *Z*-stacks were obtained from each field-of-view and were later imported into ImageJ. Image stacks were summarized to yield an average field-of-view for the local 20-μm section. Spinal cord sections were first assessed with epifluorescence to locate the glial-scar limits. A confocal image was taken at this location at both the rostral and caudal scar limits, and was used for the assessment of GFAP density. The maximum NF200 distance was measured from the GFAP scar limit in three adjacent sections and was averaged. Average NF200 density was assessed by counting the number of NF200 fibers >25 μm in each field-of-view.

### *Sema4D* siRNA assay

Primary cortical astrocytes (Lonza) were cultured in DMEM plus 15% bovine serum. The purity of the cultures was assayed by staining cells for anti-GFAP, anti-β-III-tubulin, anti-Olig2 and anit-Sox2. Only pure cultures of GFAP-positive cells were used for the following assays. P5 astrocytes were seeded on 24-well plates at a density of 5×10^5^ cells/cm^2^. At 24 hours after seeding, the culture medium (DMEM + 15% FBS + 1% Pen/Strep) was removed and replaced with 400 μl of media without antibiotics. siRNAs targeting *Sema4D* or a control siRNA (Ambion) were diluted in 50 μl of OptiMEM to reach a final concentration of 15 pmol per well. The diluted siRNA was mixed with 50 μl of lipofectamine solution in OptiMEM for a final volume of 1 μl of lipofectamine per well (Invitrogen), and incubated at room temperature for 20 minutes. Then, 100 μl of mixture was added to the cells, and the cells were incubated at 37°C for 48 hours. At 48 hours after transfection, a vial of primary cortical neurons (Lonza) was thawed and seeded on top of the astrocytes at a seeding density of 1×10^5^ cells/cm^2^. Co-cultures were incubated at 37°C in Neurobasal medium (Lonza) supplemented with 2% NSF-1, 2 mM L-glutamine and gentamicin/amphotericin (50 μg/ml and 37 ng/ml, respectively). The co-cultures were monitored daily to detect neurite outgrowth, and 50% of the medium was changed 2 days after seeding the cells. At 4 days after seeding, when neurite outgrowth was evident, the co-cultures were scratched with a P10 pipet tip. Phase-contrast images of the scratched co-cultures were captured at 30 minutes, 6 hours and 24 hours after scratching. Scratched co-cultures were fixed with 4% PFA at 48 and 72 hours after scratching, and then stained with mouse anti-β-III-tubulin (1:1000 dilution, Sigma) and rabbit anti-GFAP (1:800 dilution, Millipore). Images of the scratched co-cultures were captured using a Leica DMI6000B microscope. Neurite length and growth angle were measured using the simple neurite-tracer tool in ImageJ. Neurites growing directly toward the scratch were considered to have a 90° angle, whereas neurites growing parallel to the scratch were considered to have a 0° angle. The angle distribution was plotted using the Rosetta-histogram tool from Matlab. The statistical significance of the data was analyzed using a χ^2^ test.

### Statistical analyses

All results were presented as mean ± s.e.m. unless otherwise stated. Analyses were performed using Microsoft Excel or GraphPad Prism. Dataset means were compared using ANOVA for three or more tests. When two groups were compared, a Student’s *t*-test was used. Differences between groups was considered significant at three different levels (*P*-values of <0.05, <0.01 and <0.001) and are indicated in the figure legends.

## Supplementary Material

Supplementary Material
