# Supplementary material for: Precise control of miR-125b levels is required to create a regeneration-permissive environment after spinal cord injury: a cross-species comparison between salamander and rat
Source: Dis Model Mech. 2014 Apr 3;7(6):601–11. doi: 10.1242/dmm.014837 (PMC4036468; doi:10.1242/dmm.014837)
Supplement: Supplementary Material [file supp_7_6_601__index.html]

Precise control of miR-125b levels is required to create a regeneration-permissive environment after spinal cord injury: a cross-species comparison between salamander and rat — Supplementary Material 

# Precise control of miR-125b levels is required to create a regeneration-permissive environment after spinal cord injury: a cross-species comparison between salamander and rat

## DMM014837 Supplementary Material

**Files in this Data Supplement:**

- **Supplementary Material**
