# Supplementary material for: Precise control of miR-125b levels is required to create a regeneration-permissive environment after spinal cord injury: a cross-species comparison between salamander and rat
Source: Dis Model Mech. 2014 Apr 3;7(6):601–11. doi: 10.1242/dmm.014837 (PMC4036468; doi:10.1242/dmm.014837)
Supplement: Supplementary Material [file supp_7.6.601_DMM014837.pdf]

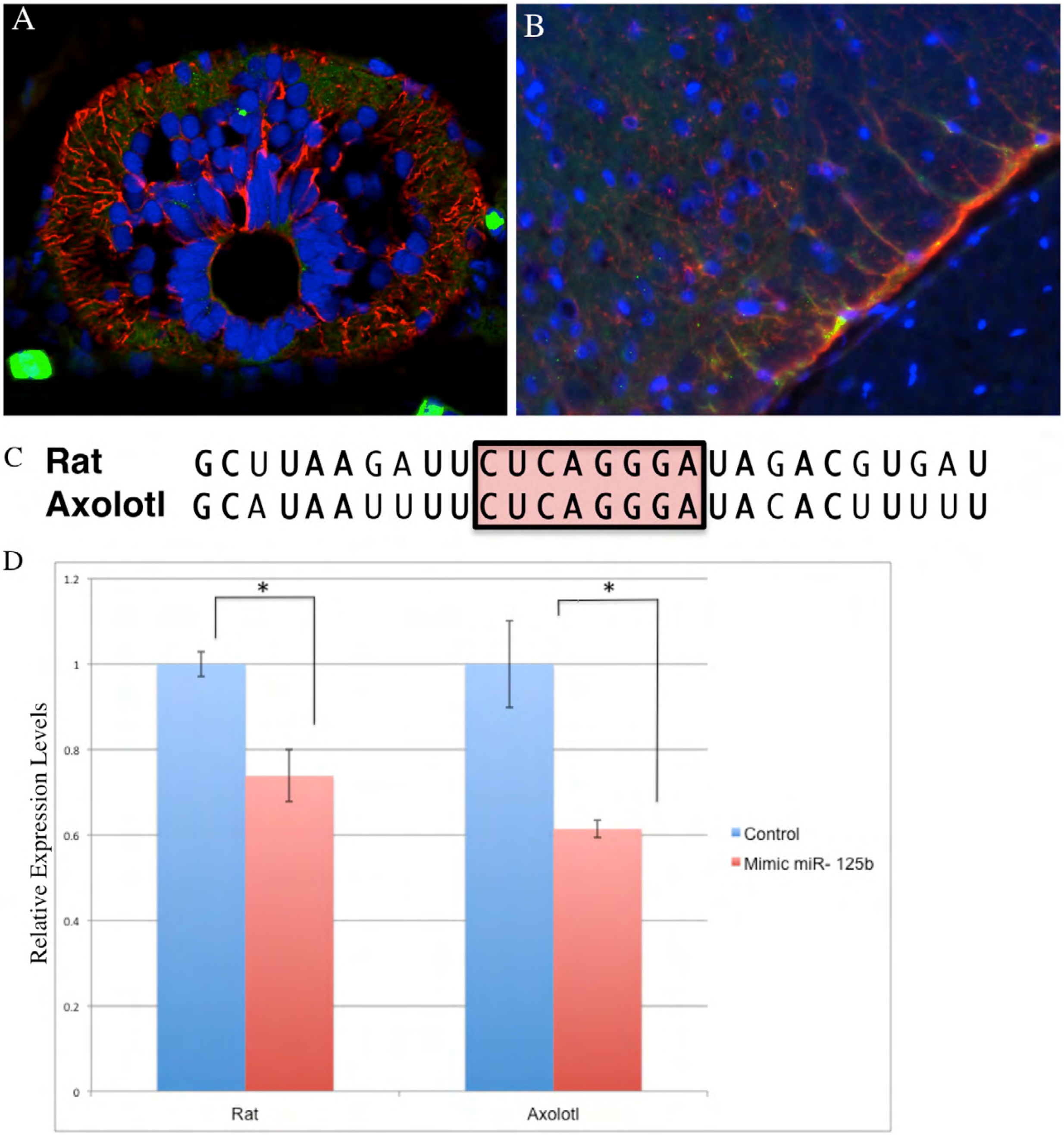

Fig. S1. (A) No Sema4D protein is detected by immunofluorescence in the uninjured axolotl spinal cord. (B) In uninjured rat spinal cord, Sema4D protein is detected in *GFAP*- positive cells. (C) The seed sequence for miR-125b is highly conserved among species, and is identical in rat and axolotl. (D) Luciferase assay shows functional targeting of miR-125b to the 3′UTR of rat and axolotl *Sema4D* (P<0.05).

GAAAGAGTGCGACAGGGCTGAGAGGGCCTGTCTGACCCGCCTGGGAGAGAGCCTACAGC
CCCGGCCGCCATTCCACGGGCTCCGGCCGCGGCGGCACCTGTTACTGGAGGCACCGGGCT
GTGTGTGGCGGGGCAGCTGCCACCGCAGGGTTCTGCTGCTGTTACTCAAACGGGACCTTC
ACTCTGCAAAAAGAGCGTGCAGAAGTTCCAGAAGAGCCCTACACGTGAAGAGAACCTGC
GTTACTTCTGAGATTAACTCTTTAAGCTTTCAGGTTCATCGCTCAGCTCAACGATGGAGAG
GAGTTTTTGTGGAGACTGACGAGCAGAAGTTTGCCGGCTGTCACAGTCGGAAATCGTGTG
GTTCTTGTGCGCGAGCAGTGACTAGGGCCGGAAATGACTACACCCCCACGACTGTAGGCT
GAGGCTGATGGTTAGCAGCTTCCCCCTGAGCGCTTCTTTAGTCTCTCTTGACACCAGCGGA
CATGCCTGCCGCCTAAAACGCGCTCTGCCTTTACTGCCCC**ATG**AGCAGCTGTTGCCGCAGT
CACTCCAGAGGGTGCGCAATGGCTCCGGCTGTGCTGTGTGCAGTCTTGGGGTTGGTACTTG
GGATAGCAGCCGCCTTTGGCCCCGTGCCCAGGACTACGTGGGAACAGCAAGAAATTCATC
TGAAACATTTTCAGGAGTCCACAGTCTTCAACTACTCAACGTTATTGCTCAGCGAAGAAAC
CAATATTTTATACATAGGCGCACGAGAAGCCATCTTTGCTGTAAATTCTTTAGATGTGTCC
GAAAAGCAGAATAAGGTGAAGTGGGCTGTTAGCGAAGACACCAAAAAAGAATGTTCCAG
AAAAGGAAAGTCCATACAGACCGACTGCCTTAATTATGTCCGTGTCTTACAACCACTAAA
TAGCAGTGTTCTGTACGTGTGTGGAACTAACGCTTTTCAACCTACCTGTAACTATTTGAGC
GTTTCTCCTTTTCAAATGGTGGGTAAGAGTGAAGATGGTAAAGGACGCTGTCCCTTTGATC
CAGCGCATAGTTACACCTCAGTCATGGTCGATGGCGAACTCTATTCTGGAACATCTTATAC
CTTTTTGGGAAGTGAGCCGATCATTCTGAGACATTCACAGCATGGTCAACTAAGAACAGA
ATATGCGGTACCTTGGCTGAACGAACCCAGATTTGTTTTTCTGATGTGATCCGAGAATCT
CCGAGTCGGGGCGGTGATGATGATAAAGTATACTTTTTCTTCACTGAAGTGTCGGTGGAAT
ATGAATTTGTTAATAAGCTGTTGATCCCCAGGATCGCCAGAGTTTGCAAGTGCATAACTAG
CCAAGCCAAGGCATTGAACTACAATACCTCCCTGGATTTACCAGACAAAACTCTTCAGTTT
GTGAAAGACCACCCTCTGATGAATGACTTGGTGATGCCAATAGGAAACCGACCCAAATTG
ATGAAGCAGAATGTGAAATACACTCAAATTGTGGTTGATAGGGTGAAAGCCCTTGATCAT
AATGTGTATGATGTCATGTTTATCAGCACAGATAAAGGAACACTTCAAAAAGCGCTCAGT
TATGCAAATGAAATGTACATCATTGAGGAAATTGAGCTTTTCCCGAATTCCGAGCCGATCC
AGACACTGATCCTGTCATCCGAAAAGGATAAGAAATTCATTTATGCAGGCTCCAATGTTG
GGGTGGTGCAGTCACCTGTTGCGTTCTGTGAGAAGTACGGCTCCTGTGTGGACTGTGTGCT
CTCTAGGGATCCCTACTGTGCTTGGCAGCAAGATAAAGACTCCTGTGTTCACATTCCCCAA
TCAGGCAGTATTGACAGTTCCTTGATACAGAAATTAAATGGGAATGCCTCCACTTGCTTGA
ATAAAAGCAAAGAGAGAGAGCTGCTTCACATCCTGAATCTCGGAAGCTCTGTAAAACTGA
CATGCTCTCAAAAGTCAAATCTAGCAACCGTTTTCTGGATGTTTGAGAGAGACCGAGTAA
CAGCTGAGGGGCCTAAGTATTATCGTCTGGGAGAAGCATTGTTCGTCTTCAATGTAACCGT
GGAGGACATGGGAGTTTATCAGTGCTTGTCTGAAGAAGATGTGTTGAATATAAAGTACTA
TCAAGTACTTGCTCAACATGTTTTGGAATTGAGAAAAGTTCCACCAACGACGACGACAGT
GGGTCCCACCCACGTTGCTACACAGACAGAAGGTAATAATGATAGATCAAAAATGTCAAC
TATGGGAACTGAAGGGTCTTCCACTCAGACATCAGCTTCCCCGACAGTACCGCTAACCAC
CACCTCCACCACCACCACAGTCATAACAAATCCAACCTCCCATGAATTAACAAGCAAGGC
CTCCATAACGGAGATCTTCAGTGCAGCTCCTGAAATTCGTTTGGAAAAGACTTTGTACTTG
ACAAAGACAAACGACAACCCCCTTTTCATGTATCTGTTTGTGTTTTTTTTTGTACTGTTTGC
GTGCCTGCTTACCTACAATTGTTACAAAGGGTTTCTGCCAGAAAATTGTCTTAAGTATCGC
TCGGCCATGTTGCCTGGAAAGGAARAAGCTGTGGCTGATTTTGTGGATTGCGAGCCGGGC
TTAAAGGAGACTTTGGTGGAGCAGAAGATTGCCCAGAGCCAAAATGGTGAGCAGCCAAA
GACATCTCGTGACACTGGCTACGAGACTGAGACAGAATGCGGGAATGGCAATATCCCACA
TAAGCATGAGACAGAGACGATAGAGGAGGATGACAGTGAAAAGCCTTYTGATGTAAACT
GTGATCTAAAATTTGCAGATTCAGATGCAGAGGGTGACTGAAGCAACATCTGTCATTCCC
CTTTGGGTATATTCAACTTTTGARKYRMATWCCYKYSRSCRMYWWSKSKKYCKGRRSCAY
RSRWMSWTGGACTGCATTGTGCCTAACTCACAACATCCTTGAACTATCTTTAACTCGCCTT
ACGTTAAAGAAAAAAGTTACTTTGCAATCCCCTCAATTCAGAGTCTTAAAATAATCTTTTT
TTAATGCATTTTTTTTGTCCGTGATTTTTGTGACCAATTTATGTTCTTCACGTGACCCAAAT
TGGTCCCTTTAGTTCCTYAGTATTTCATGCTTTATTGAATGCGGYACATTCTGTCAGCACTC
TTTACATCAGTCAAAAAGAGAAGTCGAACTTGCGTTTAATTCCAAATTGGAAATTAAATTT
AGTTTATCAGATTGGTAAGTGATTAGTCTATTGTCCAAATTAGGACAGCTTTGCCTTTAGT
GTTCTCTACTGAATGTGATTGTTTCTCTTTGAGGTTTTACTTTCTTCTGATATGATTTTTACA
CTGGTGCAAATGTAGCTAAATTTGCATAATTTT**CTCAGGGA**TACACTTTTTCCTTTTCGTT
AGAAACTGGCCACTTTTTTGTATGCAAGAGGGATGTGTGTGTGGGCTCGAAAGAGGGGAG
CACAACTAAAAAAAAAAAAAAAAAAAAAAAA

Fig. S2. Full-length axolotl sema4D. The coding sequence is marked in red, the start site ATG is highlighted in bold. The seed sequences of miR-125b is highlighted in green.

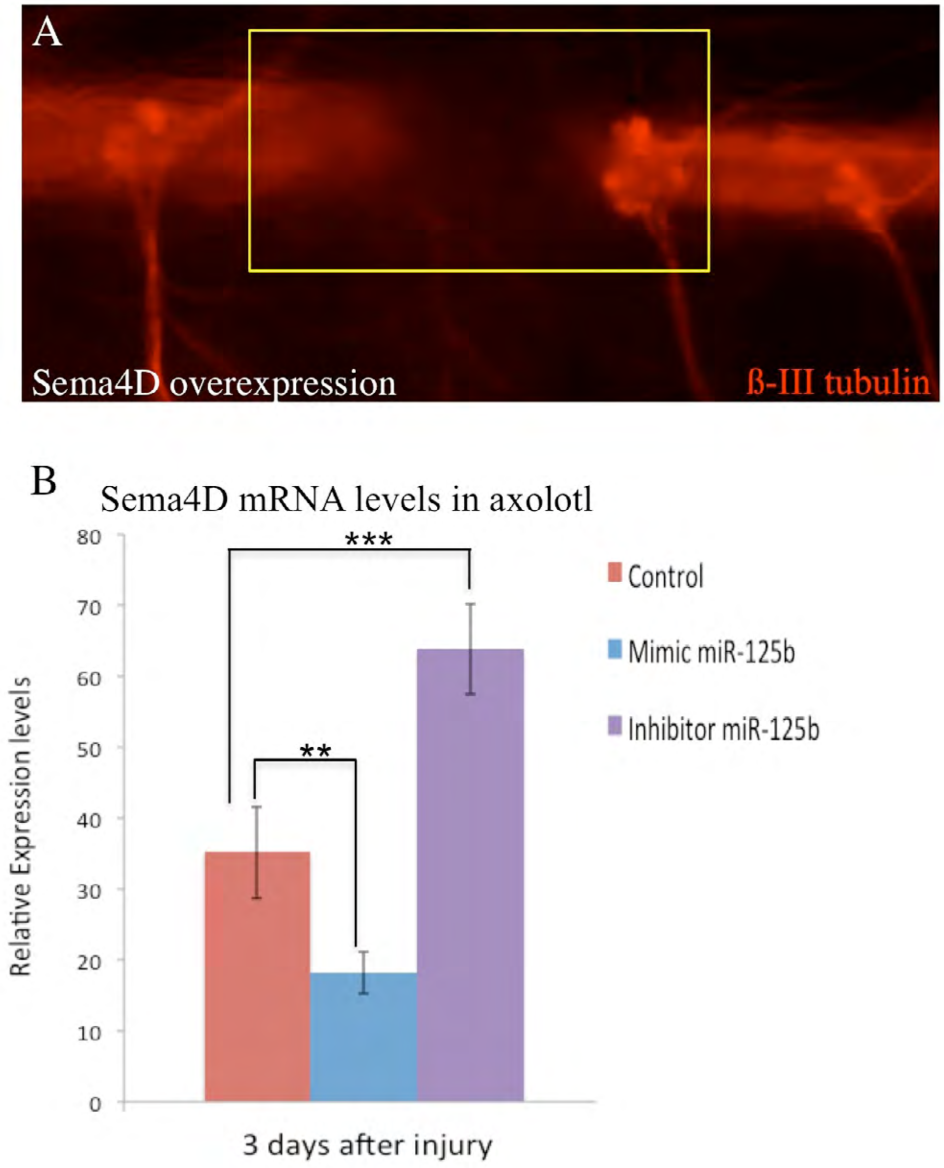

Fig. S3. (A) Wholemount anti-β-III staining on axons 7 days post injury in animals injected with a plasmid driving expression of *Sema4D* in cells at the injury site in axolotl after injury suffices to inhibit axonal regeneration. (B) Quantitative RT-PCR of *Sema4D* levels in axolotl after treatment with miR-125b mimic or inhibitor. In mimic-treated animals, *Sema4D* levels decrease ($P<0.01$); in miR-125b inhibitor-treated animals, the levels of *Sema4D* increase ($P<0.001$).

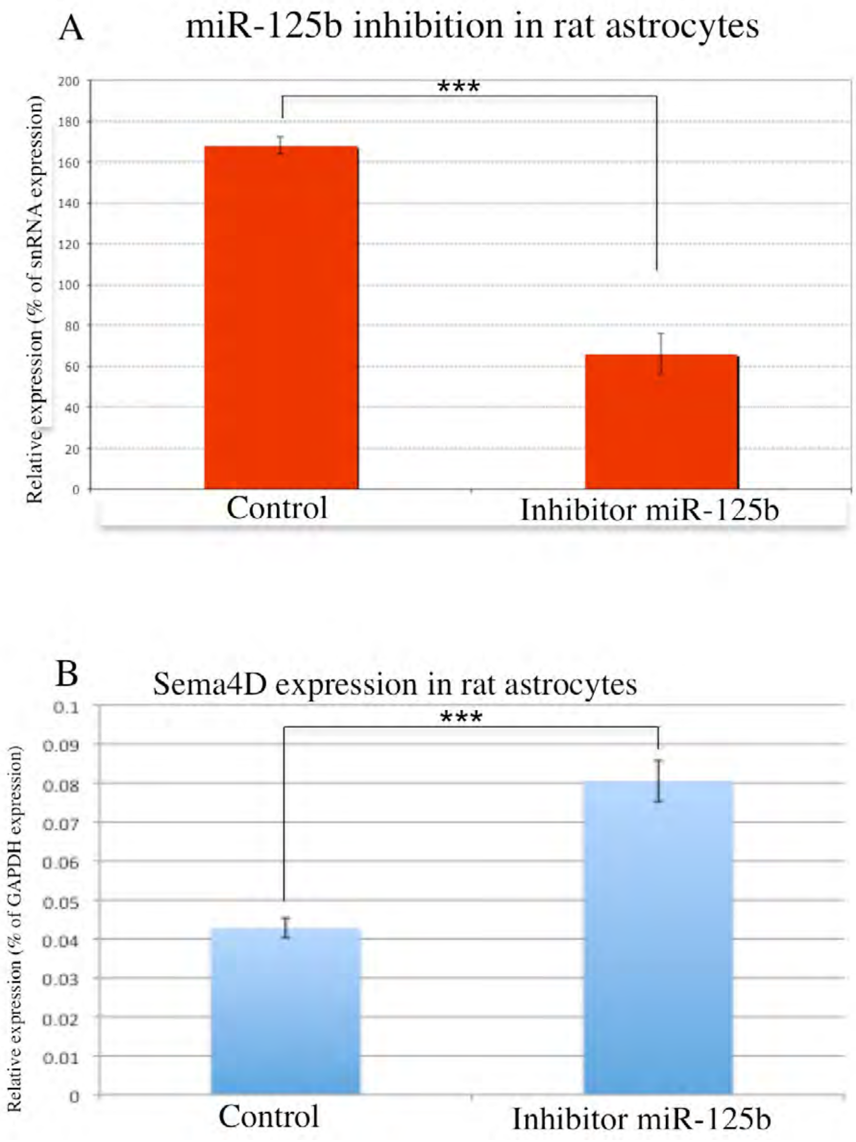

Fig. S4. (A) Quantitative RT-PCR of miR-125b in primary astrocytes after treatment with miR-125b inhibitor. (B) *Sema4D* levels increase in miR-125b inhibitor-treated astrocytes, $P<0.01$.

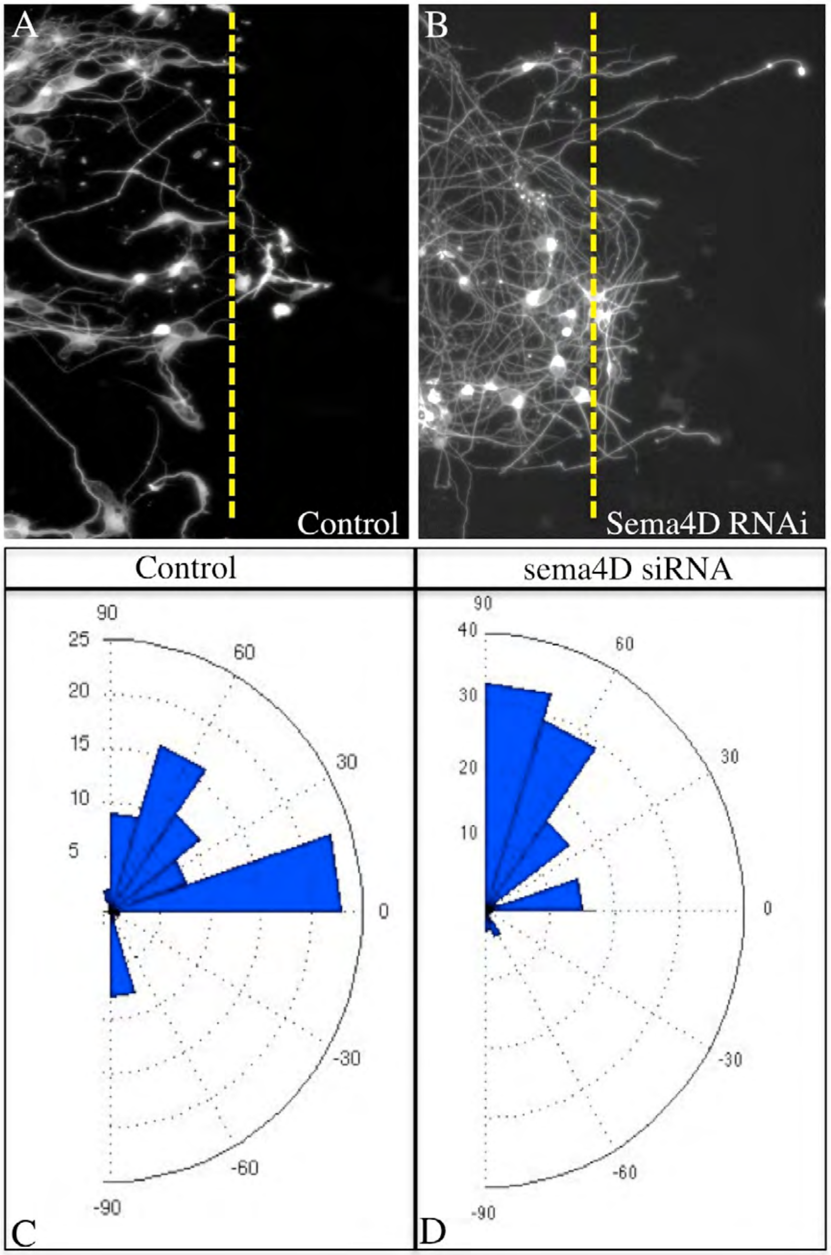

Fig. S5. Co-culture assay of primary rat astrocytes and neurons, neurons are stained with anti-β-III tubulin (A,B). In control samples after a scratch assay is performed neurons fail to regrow axons into the injury area (A). Levels of *Sema4D* are decreased in astrocytes using siRNA-mediated silencing, which creates a more permissive environment for neurons to extend axons into the injury site after a scratch assay (B). (C,D) Quantitation of the angle of projection of axons after injury in control versus *Sema4D* siRNA conditions. The angle distribution was ploted using the Rosetta histogram tool from Matlab, and the statistical significance of the data was analyzed using a one-way Anova (P<0.001).

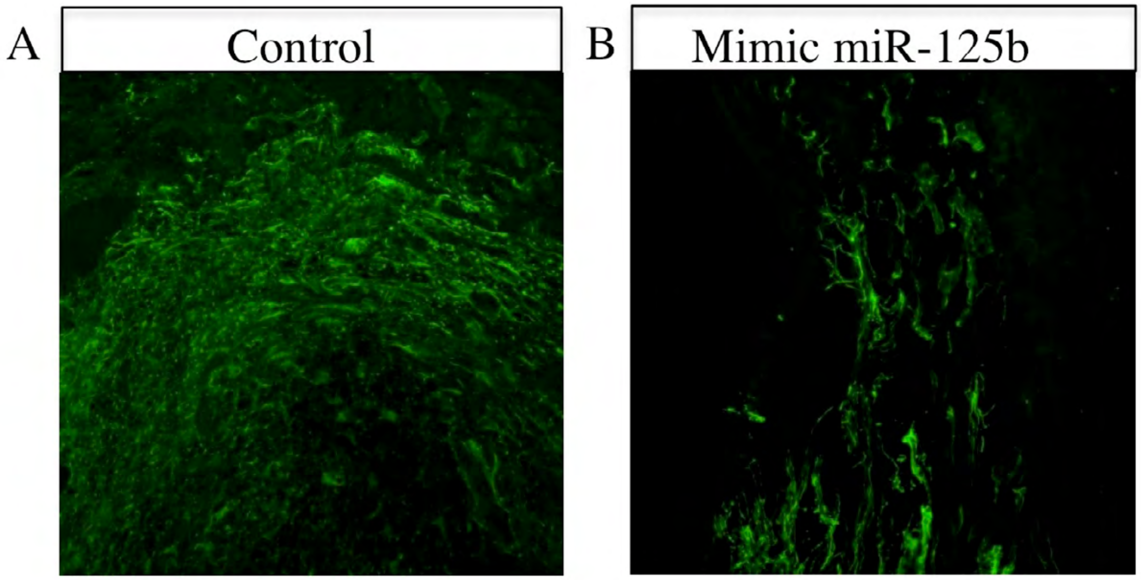

Fig. S6. (A,B) Levels of GFAP protein are significantly decreased in rats treated with a mimic of miR-125b (B) compared to the levels observed in control (A).

Supplementary Table S1

Down-regulated miR-125b target genes

Gene	Location	Family
ADAMTS4	Extracellular Space	Peptidase
ATOH8	Unknown	Other
AZI2	Cytoplasm	Other
BRCC3	Nucleus	Enzyme
DUSP7	Cytoplasm	Phosphatase
EDC3 (includes EG:315708)	Cytoplasm	Other
ESRRA	Nucleus	Ligand-dependent nuclear receptor
HOXD9	Nucleus	Transcription regulator
LETM1	Cytoplasm	Other
LRPAP1	Plasma Membrane	Transmembrane receptor
MGAT4A	Cytoplasm	Enzyme
PPP2R1B	Unknown	Phosphatase
PROS1	Extracellular Space	Other
RALGPS2	Unknown	Other
RASGEF1A	Unknown	Other
SBNO1	Unknown	Enzyme
SESTD1	Extracellular Space	Other
SLC16A6	Plasma Membrane	Transporter
SLC44A2	Extracellular Space	Transporter
SNX33	Cytoplasm	Other
TBX4	Nucleus	Transcription regulator
TSPAN8	Plasma Membrane	Other
USP46	Unknown	Peptidase

Table S1. Table of downregulated genes that contain seed sequences for miR-125b in their 3′UTR from the array on mimic-treated rats.
